# Social ideological influences on reported food consumption and BMI

**DOI:** 10.1186/1479-5868-5-20

**Published:** 2008-04-16

**Authors:** Wei C Wang, Anthony Worsley, Everarda G Cunningham

**Affiliations:** 1 School of Exercise and Nutrition Sciences, Deakin University, Melbourne, Australia; 2 Victorian Health Promotion Foundation, Honorary professor, School of Exercise and Nutrition Sciences, Deakin University, Melbourne, Australia; 3 Faculty of Higher Education, Swinburne University of Technology, Melbourne, Australia

## Abstract

**Background:**

The purpose of this study was to investigate relationships between ideological beliefs, perceptions of the importance of health behaviours, health attitudes, food consumption, and Body Mass Index (BMI). A behavioural model was hypothesized based on the Theory of Reasoned Action (Fishbein & Ajzen, 1975).

**Methods:**

A survey was conducted among shoppers aged between 40 and 70 years at Eastland Shopping Centre, Melbourne, Australia. The hypothesized model was tested with this empirical data (*n *= 410) for younger (*n *= 151) and older (*n *= 259) age groups using structural equation modelling.

**Results:**

The findings generally support the study hypotheses. For both groups, egalitarianism had a direct and positive influence on perceptions of the importance of health behaviours. Materialism and masculinity impacted negatively on health attitudes, which positively influenced importance of health behaviours. Perceptions of importance of health behaviours impacted positively on the consumption of healthy foods such as vegetables and fruits, but negatively on consumption of unhealthy foods including sweets and fats. However, BMI was significantly influenced by the consumption of unhealthy foods (e.g., sugar and fats) only for the younger age group. Hence, the associations between beliefs, attitudes, consumption behaviours, and BMI outcomes differed between younger and older age populations.

**Conclusion:**

Social ideological beliefs appear to influence health attitudes and thereafter, the consumption of healthy and unhealthy foods and BMI via different pathways.

## Introduction

Current demographic trends indicate the presence of ageing populations in most industrialized countries [1]. The ageing population has been linked to the post World War II baby boom [2]. Baby boomers are classified as those born between 1946 and 1964 [3]. This generation has had a profound impact on society and the economy throughout all stages of their lives [4]. For instance, it is anticipated that as the baby boomers age, there will be a substantial increase in the number of people aged over 65 years after 2010. As such, it will be imperative to prevent or ameliorate exposure to ill-health conditions and avoid associated medical costs. For example, dietary behaviour plays an important role in people's health [5], and it will be important to understand the predictors of healthy diets in this middle-aged population.

Increasing numbers of older people with poor health and disabilities will mean significant impacts on medical care costs, as well as other economic and social factors, within all industrialized countries. Lopez et al. [6] and Rice and Fineman [7] are among those who predict that these costs will increase substantially during the next 30 years as a result of the baby boomers entering their later lives. Based on these predictions, it is clearly advisable that issues relating to the health status of the baby boomer generation are addressed today, and not retrospectively when they enter their later life.

Lifestyle risk factors play an important role in people's health conditions [8-10]. Lifestyle is a loose term that describes the way a person lives, and includes patterns of social relations, consumption, entertainment, and dress [11]. Attitudes, values, or ideologies, are also reflected in lifestyle choices [12], which are associated with a sense of self identity and the creation of cultural symbols for the way a person lives. In addition, habits and reasoned actions constitute the behaviours and practices within lifestyles [13]. Given that diet is a key factor determining many health outcomes, the present study focuses on middle-aged people's food choices (one component of lifestyle practices), as well as their ideological beliefs and attitudes.

Beliefs, attitudes, and behaviours have received substantial attention in the literature and have been extensively examined in relation to health-related behaviours. Among the many theories explaining health-related behaviours are the Health Belief Model [14], Protection Motivation Theory [15], and Social Cognitive Theory [16]. Perhaps the most extensively researched attitude and behaviour models are the Theory of Reasoned Action [17,18] and Theory of Planned Behaviour [19,20]. The Theory of Planned Behaviour is essentially an extension of the Theory of Reasoned Action [21]. Both theories propose that individuals are more likely to intend to perform a behaviour if they hold favourable attitudes towards performing the behaviour [22]. Generally, behaviour is caused by attitudes and beliefs and attitudes are formed by evaluation of available information. However, these theories fail to explain the sources of beliefs which are evaluated and form the basis of attitudinal components. In this paper, we argue that beliefs are derived from broader sets of ideological views, which, in turn, emerge from previous experiences and learning, as well as social, cultural, and demographic influences.

Behaviours are influenced by beliefs and attitudes [18]. Accordingly, the world views and ideological beliefs that people hold influence their dietary patterns [23]. Research into the relationship between ideological beliefs, health, and health-related behaviours demonstrates that egalitarian views are associated with vegetarian diets [24,25]. Materialism is associated with high health-risk behaviours [26]. Individuals identified as having a "macho" orientation are characterized as being assertive, competitive, aggressive, and ambitious [27]; qualities found to increase the likelihood of less healthy behaviours [28]. So-called high "macho" individuals have been shown to be preoccupied with fulfilling needs for personal satisfaction at the expense of adopting healthy eating recommendations [29].

Given that relationships between ideological beliefs, health attitudes, and dietary behaviours remain to a large extent unexplored, a case can be made to investigate this area further. Moreover, "social ideologies" are an important element in nutrition promotion because psycho-sociological determinants of dietary behaviours are amenable to change, when compared with socio-demographic determinants [30]. For these reasons, the current study extends Fishbein and Ajzen's [18] model by including an assessment of the impact of egalitarianism, materialism, and masculinity on health attitude, the importance of health behaviours, food consumption behaviour, and Body Mass Index (BMI).

### Egalitarianism

Inequality is a prevailing social problem. According to Hofstede [31], equality (and its converse, inequality) is a multifaceted phenomenon which can occur in a variety of domains, including physical and mental characteristics, social status, prestige, wealth, power, laws, rights, and rules. In modern democratic societies, the term "egalitarian" refers to an ideology that values equality and promotes the abolition of social inequalities [32]. Furthermore, egalitarianism generally embraces principles of tolerance, fair play, compassion for those in need and respect for the rule of law [33].

Daniels et al. [34] highlighted that egalitarianism must be one of the social determinants of health. Studies have shown that health trends are associated with the degree of egalitarianism within society [35]. For example, individuals' life expectancy is largely determined by their socio-economic status: the richer and better educated people are, the longer and healthier their lives. However, this life expectancy and social class pattern only holds up to a certain point [36]. According to 2007 estimates [37], Sweden and the USA had GDP per capita of US$36,900 and US$46,000, respectively, yet Sweden has a life expectancy two years longer than the USA. The longer life expectancy in Sweden may be because Sweden is a more egalitarian country when compared to the USA [27]. Thus, it appears that egalitarianism may lead to better health at the national level. However, the relationship between egalitarianism and health at the individual level remains unknown.

Power distance is a cultural dimension typically studied in cross-cultural research, and refers to a society's level of tolerance for inequality. Hofstede [31] found that Australia had low power distance relative to other countries. In other words, Australians generally expect power to be distributed more or less equally across social groups. This indicates an expectation of equality in society, including government, organizations, communities, and families, and suggests that a high proportion of Australians hold strong egalitarian views. Furthermore, health can be effectively protected by stronger social cohesion and community life [38,39], which can only be exhibited in a more egalitarian society [40]. However, the relationships between health and egalitarian views still remain largely unexamined. For example, egalitarian views may affect health via individuals' health attitudes and lifestyles, and particularly their food choice behaviours. Therefore, the present study examines the relationships between egalitarian views, health attitudes and dietary behaviours among Australian middle-aged people. It is hypothesized that individuals who hold egalitarian views are more likely to have positive attitudes towards health and to practice healthier dietary behaviours than those who are less egalitarian.

### Materialism

Belk [41] defined that materialism is a tendency to believe that consumption of goods and services is one of the greatest sources of satisfaction and dissatisfaction in life. Belk [42] also claimed that the growth of materialism has become one of the predominant consumer ideologies in modern society, strongly influencing consumption behaviours including food choices. Worsley [43] extended this position, emphasising that materialism is related to consumption of so called, fashion foods. In recent times, Lowe and Worsley [44] found that in a Chinese population, individuals classified as having strong materialistic values or active materialistic lifestyles consumed high levels of luxury foods, such as imported biscuits and confectionery.

A number of studies [45] suggest that materialism is negatively related to psychological well-being and positively associated with unhappiness, dissatisfaction, depression, anxiety, anger, isolation, and alienation [46,47]. Furthermore, it has been claimed that materialism also leads to behaviour that is indulgent, hedonistic, selfish and vain [48]. In relation to health-related behaviour, Williams et al. [26] found positive relationships between health-risk behaviours and materialistic orientation. Eckersley [23] concluded that the more materialistic people are, the poorer their quality of life. Therefore, the present study sought to understand whether materialism influences individual's health attitudes, which, in turn, may impact on dietary behaviours. It was hypothesized that individuals who are highly materialistic are more likely to be less concerned about health and to consume less healthy foods than people who are less materialistic.

### Masculinities

The strong association between food and gender is illustrated by the division of labour in food preparation [49,50], and different consumption patterns of food type [51] and portion size [52] by men and women. Traditionally, women's roles within the family have mainly involved food preparation and the serving of food [53], while men have functioned as food providers [54,55]. Moreover, men consume more "heavy foods" overall, including meat and potatoes, while women generally prefer lighter foods such as chicken and salads [56,57]. Men also tend to eat and drink larger portions of foods and beverages compared to women [58]. Given the hierarchical and patriarchal structure typical of families in much of Western contemporary society and the tendency of women to subordinate their food preferences to those of their spouse and children [59], household food choices are often masculine food choices [60].

Among various models of masculinities [49,61], "macho" masculinity is only one, aggressive, form of masculine orientation. "Macho" masculinity refers to a composite of beliefs, values, attitudes, emotions, and behaviour patterns [62], which propose that men must be assertive, powerful, aggressive, competitive, ambitious, and independent, capable of defending their honour and rights and "showing manly superiority" [63]. These characteristics are postulated as the opposites of the traditional feminine qualities reflecting nurturance and modesty. However, masculinity and femininity are gender-related (rather than sex-related) self-concepts [64], and traditional gender ideologies have undergone some degree of transformation over recent generations. As such, it is important to emphasize that women can also be "macho" and demonstrate the need to be powerful, assertive, aggressive, ambitious and independent. The present study investigates how health attitudes and eating behaviours are related to "macho" ideology in general.

Vitell et al. [29] found that macho individuals are less likely to be influenced by rules or guidelines. As such, it is plausible that high macho individuals may focus more on personal satisfaction and subsequently disregard many healthy eating recommendations [65,66]. In this study, it is hypothesized that individuals who hold "macho" views are less likely to have positive attitudes towards health and will consume fewer healthy foods, than those who are identified as less "macho" (feminine).

Perception is defined as "to become aware or conscious of", which enables the person to interact with the information perceived through behaviours [67]. A number of studies [22,68] have demonstrated that attitudes can be strong predictors of health behaviours. Thus, perceptions and attitudes are among the many factors determining health behaviours. Furthermore, BMI is related strongly to dietary behaviours such as consumption of different types and amounts of foods [69].

The current study targeted middle aged Australians between 40 and 70 years old. People born in the 1940s and in the 1960s are likely to have significantly different life experiences. For example, people born in the 1960s may be more closely related to generation X rather than the baby boomer generation [70]. For this reason, analysis of this baby boomer sample was split into "older baby boomers", those aged from 56 to 70 inclusive, and "younger baby boomers", those aged from 40 to 55 inclusive.

**Figure 1:**
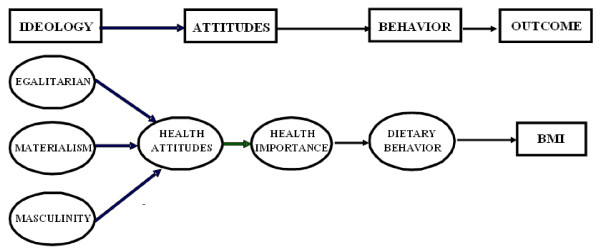
Framework of belief, attitude, behaviour, and outcome variable.

In the present study, the Theory of Reasoned Action was extended and modified with the inclusion of three sets of ideological beliefs (i.e., egalitarianism, materialism, and masculinity) as antecedents of importance of health behaviours/health attitudes, behaviours, and BMI. Figure 1 illustrates possible causal relationships among ideological beliefs, attitudes, behaviours, and BMI. In particular, it is hypothesized that ideological beliefs influence health attitudes and the perceived importance of health behaviours. Furthermore, the perceived importance of health behaviours and attitudes impact on BMI through dietary behaviours. The aim of the study is to investigate the influence of these social ideological beliefs on perceptions and health attitudes, food consumption and BMI of Australian middle-aged people.

## Methods

### Procedure

Using convenience sampling, shoppers in a metropolitan shopping centre were personally approached. Each potential participant was given a questionnaire, along with a prepaid return envelope and covering letter explaining the purpose of the study. Participants were asked to complete the questionnaire at a time convenient to them and to return the questionnaire in the prepaid envelope within the next two weeks. A total of 1000 questionnaires were distributed over a three day period. The questionnaires took approximately 20 minutes to complete. This data collection method was used because it is quick and relatively inexpensive, and because face-to-face conversation allows the researcher to effectively address questions raised by the potential participants, and results in a lower rate of refusal [71].

### Participants

The participants were 501 individuals (response rate = 50%) who visited Eastland Shopping Centre, Melbourne, Australia. Useable responses for 432 cases were included. Participants age was between 40 and 70 years (M = 57.45, SD = 8.02). Women accounted for 81.5% of the sample, which is consistent with previous food shopping surveys [72]. Using self-reported weight (Mean = 71.11, SD = 13.57 Kg) and height (Mean = 165.93, SD = 8.77 cm), BMI was calculated and resulted in a mean of 25.83 with a standard deviation of 4.57. The majority were born in Australia (72.2%) and the UK (15.5%). Forty-two percent (42.4%) of respondents had secondary school qualifications, 22.5% had a technical or trade qualification, and 15.7% were tertiary educated. These demographics closely match the characteristics of the Australian population [73], and suggests a highly representative sample.

### Instruments

#### Food Variety Instrument [74]

The Food Variety Instrument was adapted for use in this study. The original Food Variety Checklist comprises 54 items (e.g., apples, potatoes, and fish) addressing 14 food groups (e.g., fruits, vegetables, and seafood). However, there are no questions about portion size or frequency of consumption in the original Food Variety Checklist. If a food was consumed during the previous week, a score of 1 is given [75]. This instrument has been used in several studies in relation to food variety and health outcomes and demonstrated significant associations between food variety scores and health problems [76,77]. In the present study, this instrument was modified to include 60 foods across 14 food domains. In order to obtain adequate information on frequency of food consumption, the response format was also amended. The modified version of the checklist required the participants to report how often they ate the particular food (e.g., fruit or vegetable) during last week by using a 3-point scale where (0) represented 'did not eat', (1) 'ate 1–2 times', and (2) 'ate more than 2 times'. Higher scores indicate more frequent consumption of the particular food category.

BMI is strongly associated with energy intake gained from diverse amounts and types of foods [69,78]. In order to obtain approximate energy estimates, food consumption frequencies were converted to energy scores based on the energy (KJ) produced by foods per 100 (g) consumed. These energy values were derived from the FoodWorks software [79]. To calculate the energy scores, the frequency of consumption scores of 0, 1, and 2, which respectively represented "did not eat", "ate 1 to 2 times", and "ate more than 2 times" during the last week, were multiplied by the energy values for particular foods. Using "apple" as an example, if a participant did not eat an apple during the last week, the energy score would be zero for "apple". If she/he had eaten one or two apples during the last week, the energy score from consuming "apple" would be 228 KJ. However, if she/he had consumed more than two apples during the last week, the energy score produced by eating apples is 456 KJ (i.e., 2 × 228 KJ).

#### Importance of health behaviour [80]

The Importance of Healthy Dietary Behaviour instrument was adapted from Petrovici and Ritson's [80] study. It consisted of nine items describing nine different dietary health maintenance behaviours (e.g., how important to you is consuming a lot of fruit and vegetables?). A Confirmatory Factor Analysis (CFA) using LISREL 8 conducted by Petrovici and Ritson, suggested a two-factor solution reflecting positive dietary actions (Cronbach's α = .75) and negative dietary actions (Cronbach's α = .72). An item relating to alcohol moderation was removed as it acted independently. The purpose of the current study was to measure the degree of importance attributed to health dietary behaviours as perceived by the individuals. The initial response wording of this 5-point Likert scale was changed to not at all important (1) to extremely important (5), rather than strongly disagree (1) to strongly agree (5) as in the original instrument. Higher scores on any item reflect a greater perception of the importance of health behaviours.

#### Health attitudes [81]

The Health Attitude Scale contained 15 items reflecting three subscales, namely feelings regarding health (items 1–5; e.g., Practicing a healthy lifestyle is exciting), beliefs regarding disease prevention and healthy lifestyle (items 6–10; e.g., I cannot change my health status), and intention to act for better health (items 11–15; e.g., I will take care of my health). The response format was a 5-point scale rating from 1 (strongly disagree) to 5 (strongly agree). Scores on negatively worded items were reversed, with higher scores reflecting more positive health attitudes. The reliability coefficients (Cronbach's α) reported for the three constructs were .78, .70, and .81.

#### Egalitarianism scale [82]

Egalitarian views were assessed with the Egalitarianism Scale. The six-item General Egalitarianism scale (e.g., It is really not a big problem if some people have more chances in life than others) was used in the 1986 and 1992 American National Election Studies. The internal reliability coefficients were reported as .74 and .75 for the 1986 and 1992 data respectively. Ng and Burke [83] used the Egalitarianism scale in a Canadian sample and reported an internal reliability coefficient of .73. The response format was a 5-point scale ranging from 1 (strongly disagree) to 5 (strongly agree). Negatively worded items were reversed with higher scores indicating stronger egalitarian views.

#### Masculinity scale [84]

The Masculinity scale included five items (e.g., It is preferable to have a man in a high level position rather than a woman). This scale had a 5-point response format, ranging from 1 (strongly disagree) to 5 (strongly agree) with higher scores representing stronger masculine views. A Cronbach's alpha of .83 was reported for a sample of the Canadian population [83].

#### Materialism scale [85]

The Materialism Scale contained 18 items and provided subscales that assessed three materialistic domains, namely success (items 1–6; e.g., I admire people who own expensive homes, cars, and clothes), centrality (items 7–13; e.g., I like a lot of luxury in my life), and happiness (items 14–18; e.g., My life would be better if I owned certain things I don't have). The response format was a 5-point scale ranging from 1 (strongly disagree) to 5 (strongly agree). Positively worded items were reversed, with higher scores indicating stronger materialistic views. Richins [86] reviewed 44 articles that used the 18-item Materialism scale and reported that the average internal consistency alphas were .77 for success, .73 for the centrality, and .75 for the happiness subscale. These are consistent with Richins and Dawson's [85] earlier findings.

In addition to these socio-psychological beliefs, attitudes, and food consumption measures, social demographic background information including age, gender, height and weight, education levels and annual household income was also collected. Several studies [87-89] have shown that self-reported weights and heights are valid for determining associations in epidemiological studies. Therefore, based on the height and weight reported by the participants, a BMI index was calculated for all participants.

### Analytical procedure

Data were analysed using SPSS 15 [90] and AMOS 7 [91]. Maximum likelihood (ML) estimation was used in the current analyses, which assumes that the data were continuous and multivariate-normally distributed. Model evaluations were examined by chi-square statistics and accompanying significance tests, the ratio of chi-square to degrees of freedom (χ^2^/df). Goodness-of-fit indices reported are the Root-Mean-Square Residual (RMR), Root Mean Square Error of Approximation (RMSEA), Goodness-of-fit Index (GFI), Adjusted Goodness-of-fit Index (AGFI), Tucker-Lewis index (TLI), and Comparative fit index (CFI) [92]. When the models were considered to fit the data well, the following criteria were met: χ^2 ^probability p > .05, χ^2^/df < 2, RMR < .05, RMESA < .05, GFI > .95, AGFI > .95, TLI > .95, and CFI > .95.

Scale scores were derived by parcelling the items measuring the same construct or subconstruct. Item parcelling [93,94] was used to remedy non-normality and lack of continuity of the indicators to ensure that the assumptions of ML estimation method were met. One advantage of applying structural equation modelling (SEM) rather than path analysis is that measurement error can be estimated and controlled. Once composite variables have been computed through the item parcelling method, it is possible to fix both the regression coefficients, which reflect the regression of each composite variable on its latent variable, and the measurement error variances associated with each composite variable via the formulae provided by Munck [95]. Using Munck's formula, regression coefficients can be derived from SD $\sqrt{\alpha }$ and error variances from SD^2 ^(1 - α). Both fixed values can be used for single indicator construct in the structural equation model.

## Results

Prior to analyses, data were screened for missing values, accuracy of data entry, outliers and normality [96]. A non-significant Little's Chi-square statistic indicated that missing data were missing completely at random. The missing value range was from 0.5% to 14.8% for dietary data and less than 4% for the cognitive data. In this preliminary exploration, missing values were replaced via the expectation-maximization (EM) method [97]. A total of 410 cases were used in the analyses with the further exclusion of non responses on either height or weight from which BMI was calculated.

In order to establish appropriate measurement of constructs, it is necessary to recode response categories [98]. Owing to very few responses in response categories 1 and 2 for most of the items in the Health Attitude scale, the Health Importance scale, and the Egalitarianism scale, these items demonstrated negatively skewed distributions. As such, categories for these items were collapsed to form four, rather than five, response categories (scored 1 1 2 3 4). Similarly, relatively few responses on categories 4 and 5 for the items in the Materialism scale and the Masculinity scale resulted in positively skewed distributions, and the categories for these items were collapsed to form four, rather than five, response categories (scored 1 2 3 4 4). A number of items showed evidence of misfitting with the scales, suggesting that responses to these items were more random than expected for the model. Therefore, some items were removed from the scales. The remaining items were deemed to measure the appropriate constructs. Table 1 presents the means, standard deviations, skewness, and kurtosis statistics for the composite variables, as well as their corresponding internal consistency reliability values for the psycho-socio belief and attitude measures.

**Table 1:** Mean, standard deviation, skewness, and kurtosis for each composite variable and corresponding Cronbach's α for psycho-socio belief and attitude measures

	Number of items	Mean	Standard deviation	Skewness	Kurtosis	Cronbach's α
Egalitarianism	5	2.54	.64	.29	-.39	.56
Materialism						
Success	6	2.09	.63	.22	-.43	.68
Happiness	5	2.11	.76	.25	-.69	.77
Masculinity	4	1.81	.81	.77	-.34	.84
Health importance	5	3.31	.58	-.76	.10	.72
Health attitudes						
Belief	5	3.10	.65	-.26	-.82	.63
Intention	5	3.25	.59	-.50	-.57	.66

An exploratory factor analysis (EFA) with principal axis factoring and direct oblimin rotation was conducted on energy scores derived from consumption of different foods. The EFA clearly suggested two factors: healthy foods (e.g., fruit, vegetable, legume, seed, and fish) and unhealthy foods (e.g., sweet, oil, and meat). Although meat has been considered as an unhealthy food in many epidemiology studies [99,100], other studies recommend meat as a component of a healthy diet, not to be consumed in excess but balanced by vegetable consumption [101,102]. Because the role of eating meat in a healthy diet is unclear, it was removed from the analyses. The EFA was re-run and the factor scores of healthy and unhealthy foods were saved for use in further SEM analyses.

Table 2 shows the means and standard deviations of self-reported weight, height, and calculated BMI for the younger, older, and whole groups. Although the older group had a mean BMI of 26.04, which is slightly higher than the younger group with a mean BMI of 25.45, an independent groups T-test (*t *(408) = -1.26, *p *= .21) found that the difference in mean BMI for younger and older participants was not significant.

**Table 2:** Means and standard deviations of weight, height, and BMI for younger, older, whole groups

	Weight (kg)		Height (cm)		BMI (kg/m^2^)	
	M	SD	M	SD	M	SD
Younger group (*n *= 151)	70.81	13.34	166.84	9.42	25.45	4.47
Older group (*n *= 259)	71.28	13.72	165.41	8.35	26.04	4.62
Whole group (*n *= 410)	71.11	13.57	165.93	8.77	25.83	4.57

Note: M = mean; SD = Standard Deviation

Table 3 displays the fit statistics across the younger and older age groups. An inspection of Table 2 suggests that the proposed model fit well for both younger and older age populations, as indicated by non significant chi-square statistics, χ^2 ^(29) = 33.70, p = .25, and χ^2 ^(29) = 29.17, p = .46, for both younger and older age groups respectively. All the other fit indices were in the desired range. Therefore, it can be concluded that relationships in the data among ideological beliefs, importance of health behaviours, health attitudes, and energy score derived from consuming healthy foods (e.g., vegetable and fruits) versus unhealthy foods (e.g., oil/fats and sweets) were consistent with the hypotheses.

**Table 3:** Fit statistics for younger and older age populations

Group	Chi-square	df	*p*	RMR	GFI	AGFI	TLI	CFI	RMSEA
Younger (*n *= 151)	33.70	29	.25	.03	.96	.93	.97	.98	.03 (.00, .07)
Older (*n *= 259)	29.17	29	.46	.05	.98	.96	.10	.10	.01 (.00, .05)

**Figure 2:**
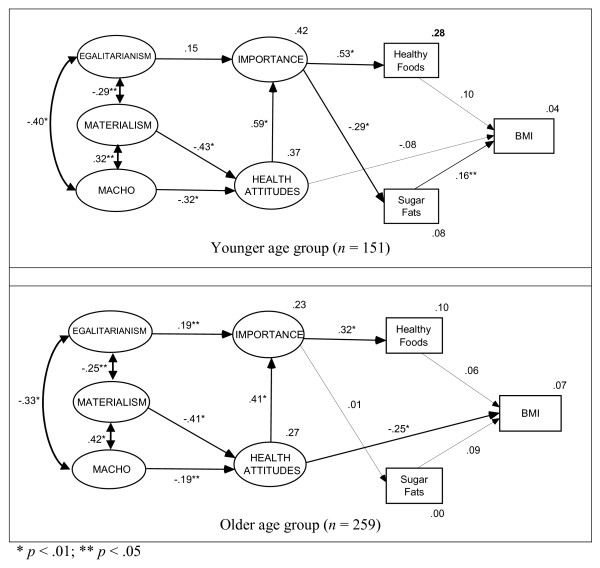
Standardized parameter estimates for the younger and older middle-aged populations.

Figure 2 illustrates the structural equation model with standardized parameter estimates for the two age groups. Noticeably, the magnitudes of the path coefficients are different across the two groups. In particular, the importance of health behaviour significantly influences sugar and fat consumption in younger age population but not for the older group. Conversely, health attitude has significantly negative impact on BMI in the older age group but not for the younger group.

Overall the findings support the study hypotheses. Specifically, Egalitarianism had a direct and positive influence on perceptions of the importance of health behaviours. Materialism and Macho orientation impacted negatively on health attitudes, which positively influenced importance of health behaviour. Perceptions of importance of health behaviours impacted positively on the consumption of healthy foods such as vegetables and fruits, but negatively on the consumption of unhealthy foods including sweets and fats. However, BMI was significantly influenced by the consumption of unhealthy foods (e.g., sugar and fats) in the younger age group. The associations among beliefs, attitudes, consumption behaviours, and BMI outcomes differed between younger and older age populations.

Consumption of unhealthy foods, including sweets and fats, was significantly and negatively influenced by the perceptions of importance of health behaviours for the younger age group. In other words, people aged between 40 and 55 years who valued the importance of health behaviours reported less consumption of sweets and fats. However, this relationship was not found within the older age group. Similarly, BMI was found to be significantly affected by health attitudes for the older age group. In other words, people aged between 56 and 70 years with better health attitudes had lower BMI. In contrast, this association was not demonstrated in the younger age group. The positive association between consumption of sweets and fats and BMI was also greater in younger group than in older group.

## Discussion

The present study extends the Theory of Reasoned Action by including ideological beliefs in the model. This model suggests that perceptions of importance of health behaviours and health attitudes are determinants of food consumption behaviours. These perceptions and attitudes are, in turn, influenced by specific ideological beliefs. These psycho-sociological determinants of dietary behaviours are potentially more amenable to change than socio-demographic determinants [30]. Thus, the baby boomers can be segmented according to their ideological beliefs. Accordingly, nutrition promotion could be geared for particular "ideological" target segments e.g., "macho" oriented individuals can be cultivated towards the less macho, perhaps acquiring better healthy eating habits.

The present study suggests that certain associations among socio-psychological beliefs, attitudes, food consumption, and BMI are dissimilar between younger and older age groups. For example, among people aged between 56 and 70 years, positive health attitudes are associated with lower BMI. In order to reduce BMI, better health attitudes seem to be important within the older age group. In contrast, positive perceptions of the importance of health behaviours are related to low consumption of sugar and fat among people aged between 40 and 55 years. Thus, with overweight individuals in the younger age group, health education programs may be designed to focus on enhancing consciousness of health behaviours to decrease the consumption of sugar and fat.

The findings should be viewed cautiously in the light of the study's limitations. First, the current study sample consisted of over 80% women, which reflects the fact that that women play key roles in food shopping. Future studies could consider using random samples to examine whether the proposed model differs across gender lines. Second, the Food Variety Instrument was employed partly to minimize response burden. Information concerning the portion size of food intake was not sought and information regarding foods consumed more than 2 times a week was not captured (i.e., no difference was assumed between3 and 10 times or more of foods consumed during theweek). Thus, the relationship between consumption ofvarious foods (e.g., healthy, sugary/fatty) and other factors (e.g., BMI) could be underestimated using the energy scores derived only from food frequencies of "none", "1–2 times", and "more than 2 times". Finally, it should be noted that the structural models (see Figure 2), explained only a small proportion of the variance in the outcome variables, healthy foods, sugar/fats, and BMI (i.e., less than 28% across two groups). This is consistent with previous studies utilizing the Theory of Reasoned Action and Theory of Planned Behaviour models [103,104]. Obviously, food choice is influenced by multifaceted and interrelated determinants. This study investigated only a few possible socio-psychological influences. For example, BMI is influenced by other important lifestyle factors such as physical activity, which were not measured in the present study. It is also possible that potential biases in self-reported ideological beliefs, perceptions, attitudes, and consumption patterns could have influenced the predictive power of the proposed model, opening avenues for further research in this area such as replicating the model with different populations and instruments.

## Conclusion

Social ideological beliefs appear to influence the perceived importance of health behaviours and health attitudes, which in turn, impact on the consumption of healthy/unhealthy foods and BMI via different pathways in younger and older middle-age groups. These ideological beliefs could form the basis for segmentation of the baby boomer population, which may bring about more effective nutrition promotion in this age group.

## Competing interests

The author(s) declare that they have no competing interests.

## Authors' contributions

WCW performed the data collection and the writing of the manuscript. AW provided advice on the survey design, acquisition of data, and the construction of the manuscript. EGC assisted with the statistical analyses and commented on the manuscript. All authors read and approved the final manuscript.
